# Supplementary material for: Arbutus unedo L. Fractions Exhibit Chemotherapeutic Properties for the Treatment of Gastrointestinal Stromal Tumors
Source: Plants (Basel). 2024 Apr 25;13(9):1201. doi: 10.3390/plants13091201 (PMC11085211; doi:10.3390/plants13091201)
Supplement: Supplementary file 1 [file plants-13-01201-s001.zip › plants-2916535-supplementary.pdf]

Supplementary information

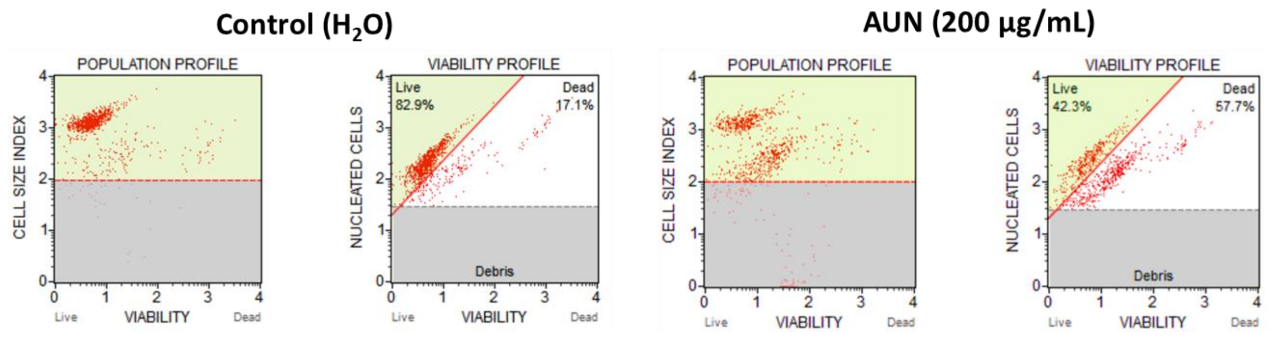

Supplementary Figure S1. Analysis of viable and dead cells in GIST-882. Cells were treated with AUN 200 µg/mL and compared to control. Dead cells are labeled using Guava® ViaCount™. Each red point represents a cellular event detected by the flow cytometer. The graph on the left defines the “Population profile” and classifies each event by the “cell size index” (x-axis) and the positivity to the “viability” dye (y-axis). Instead, the “viability profile” on the right defines the event on the positivity to the “nuclear dye” (y-axis) and “viability” (x-axis). A representative replicate of an experiment is shown.

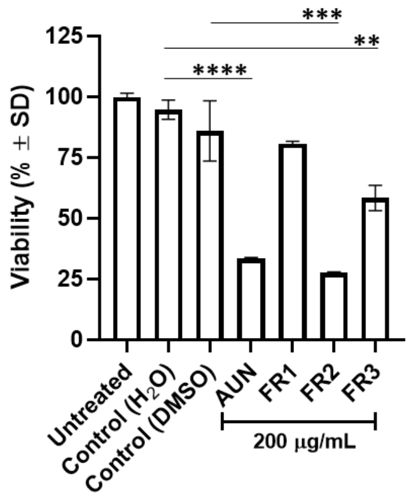

Supplementary Figure S2. Effect of AUN and its derived FRs in GIST-T1. Cell viability after AUN or its derived FRs treatment (FR1-FR2-FR3). The length of the treatment was 24h. Viability is expressed in percentage (%) ± SD with respect to the untreated. **** Adjusted P value < 0.0001; ***<0.001; **<0.01 (One-way ANOVA-Tuckey’s Multiple comparison test with respect to the corresponding solvent negative control). N=2.

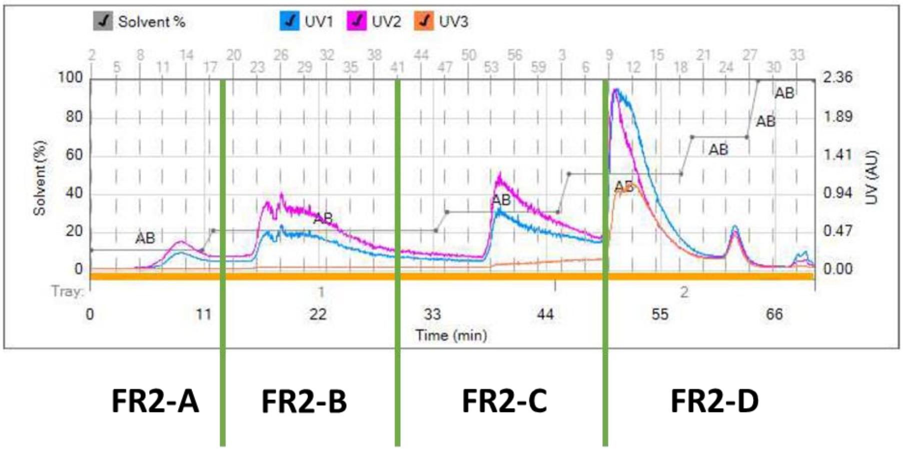

Supplementary Figure S3. Origin of FR2 subfractions through the 2nd step of bio-guided fractionation by reverse phase MPLC. FR2 was further fractionated using a reverse-phase MPLC as the most active fraction among AUN-derived fractions. Phytochemicals were eluted with different retention times by an increasing gradient of CH_3OH. The evolution of the UV absorbance at three different wavelengths (UV1=254 nm, UV2=270 nm, and UV3=340 nm) was used to differentiate four main peaks, representative of different families of phytochemicals with different solubility. Thus, FR2 was divided into FR2-A, FR2-B, FR2-C and FR2-D. FR2-A contains more polar compounds than FR2-D, which instead contains more non-polar phytochemicals that are eluted with a high retention time during chromatography.

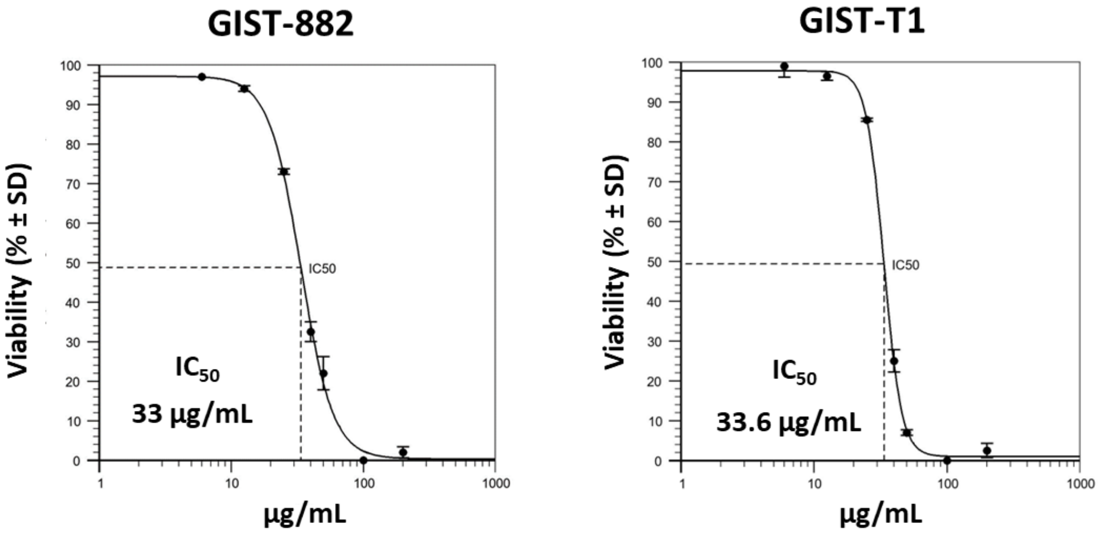

Supplementary Figure S4. IC50 Calculation of FR2-A in imatinib-sensitive GIST-882 and GIST-T1. GIST-882 (left panel) and GIST-T1 (right panel) were treated with FR2-A, and the viability was measured through Guava® ViaCount™ staining and flow cytometry. Cell viability is expressed in % ± SD with respect to control. Calculated IC50 is reported at the bottom right of both graphs.

(A)

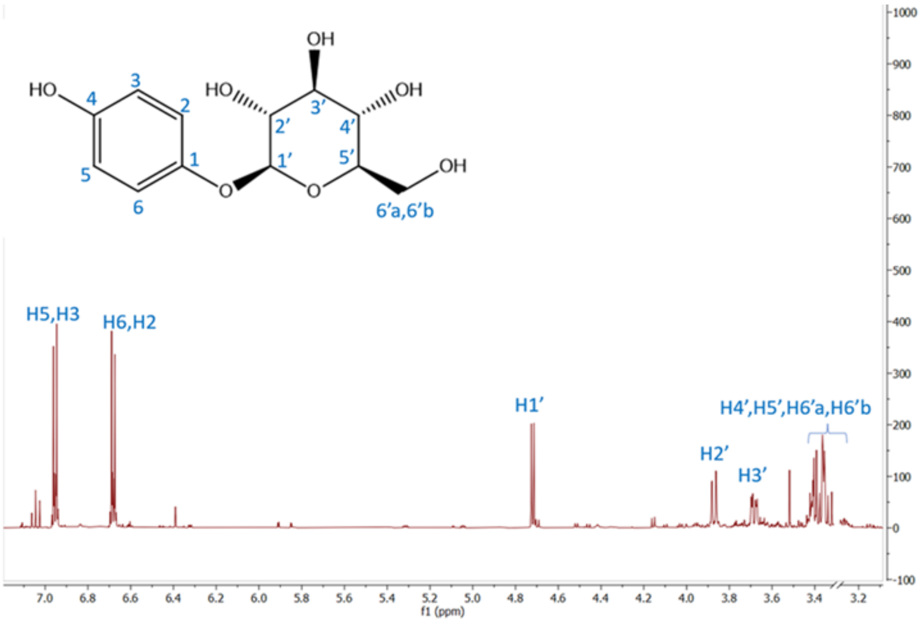

(B)

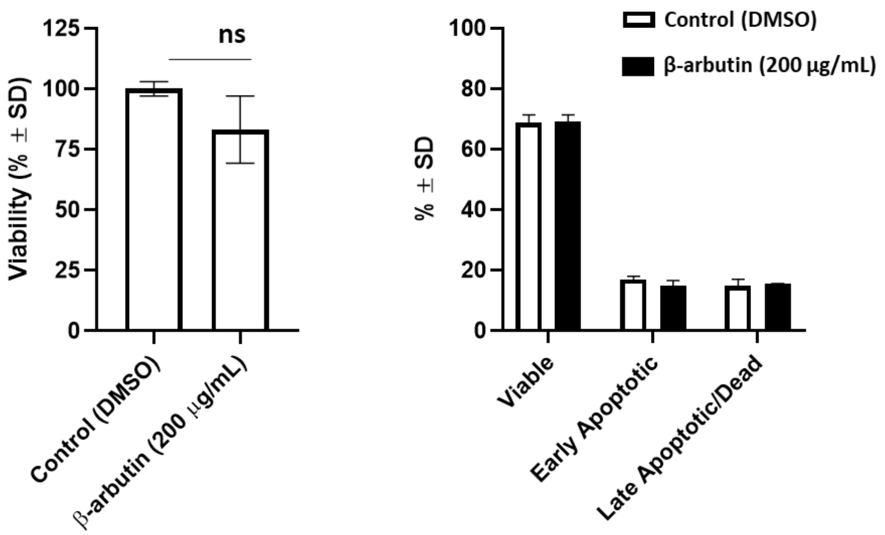

Supplementary Figure S5. β-arbutin is not the active NP of FR2-A. (A) ^{1}H NMR spectrum in MeOH-*d4* of FR2-A containing as main compound β-arbutin. Protons responsible for each spectral signal have been assigned. **(B)** Cell viability after β-arbutin treatment is shown in the left panel. The length of the treatment was 72h. Viability is expressed in % ± Standard deviation (SD) with respect to control; ns means "not significative" (unpaired t-test). The % (±SD) of living (Annexin-V (-)/ /7-AAD (-)), early apoptotic (Annexin V (+)/7-AAD (-)), and late apoptotic/dead (Annexin V (+) and 7-AAD (+)) cell populations are displayed in the right panel. N=2.

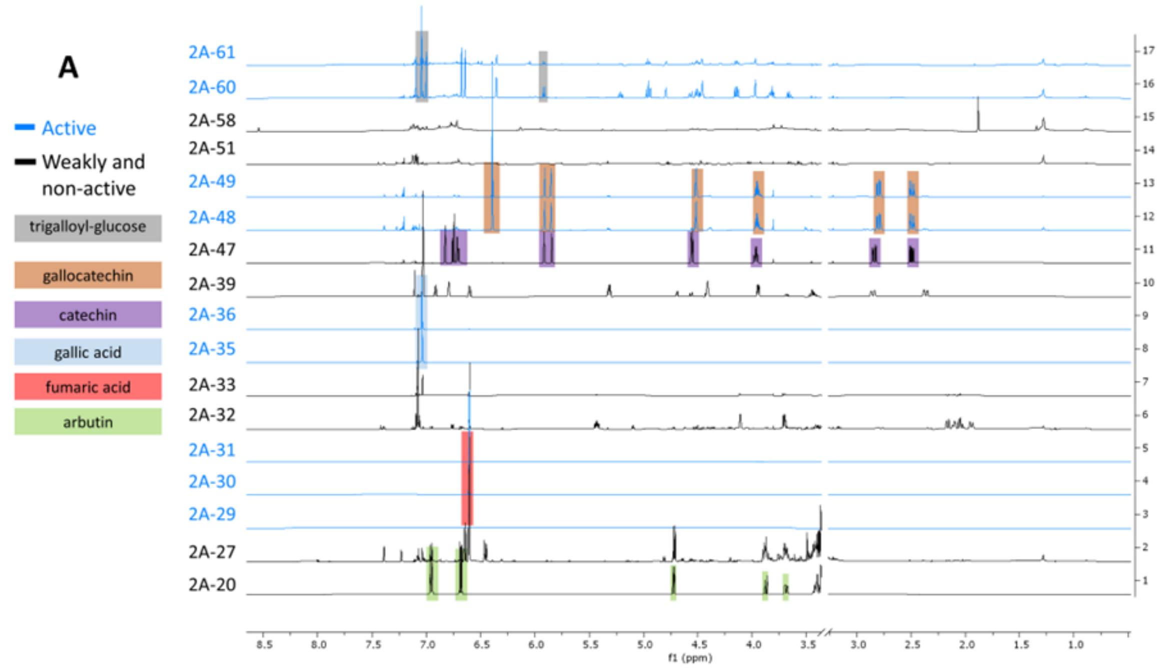

A
Active
Weakly and non-active
trigalloyl-glucose
gallocatechin
catechin
gallic acid
fumaric acid
arbutin
2A-61
2A-60
2A-58
2A-51
2A-49
2A-48
2A-47
2A-39
2A-36
2A-35
2A-33
2A-32
2A-31
2A-30
2A-29
2A-27
2A-20
f1 (ppm)

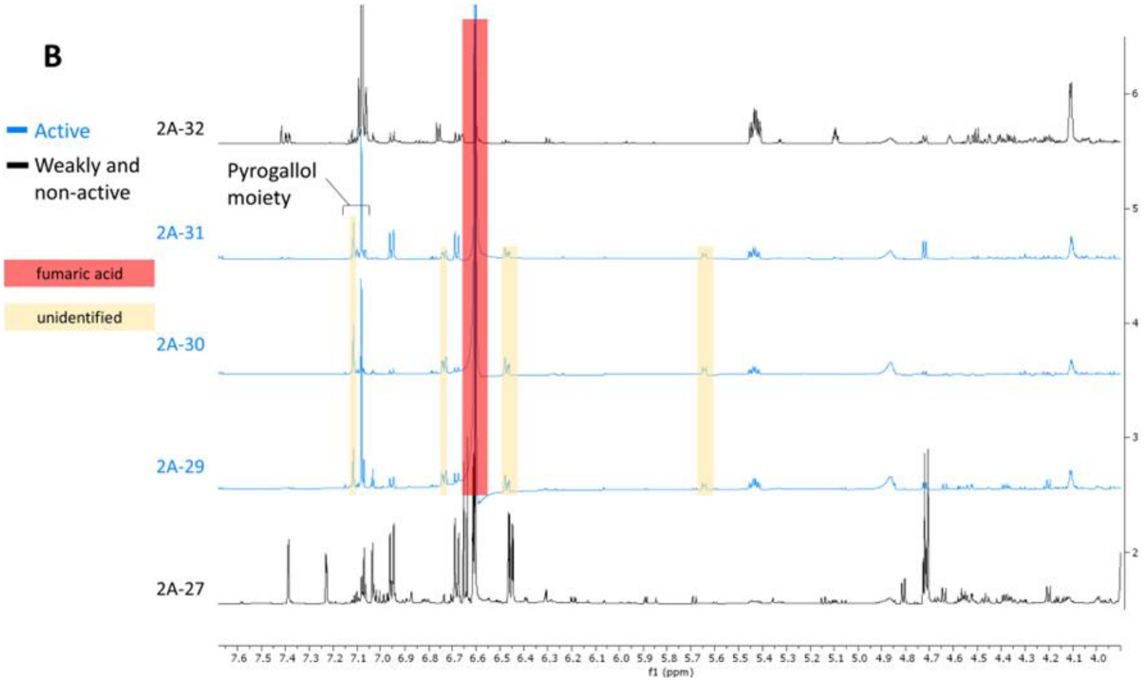

B
Active
Weakly and non-active
fumaric acid
unidentified
2A-32
Pyrogallol moiety
2A-31
2A-30
2A-29
2A-27
f1 (ppm)

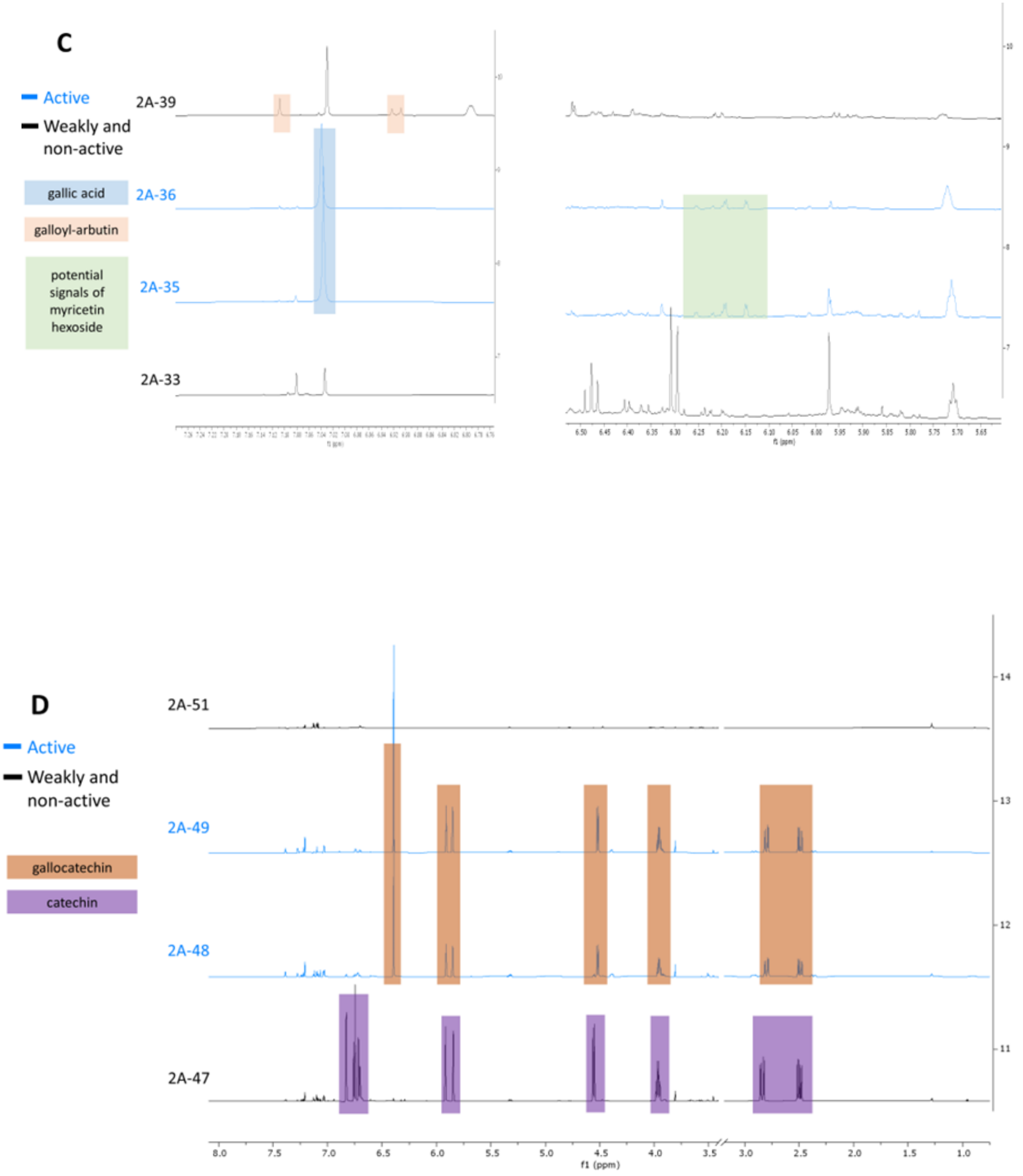
C
Active
Weakly and non-active
gallic acid
galloyl-arbutin
potential signals of myricetin hexoside
2A-39
2A-36
2A-35
2A-33
D
Active
Weakly and non-active
gallocatechin
catechin
2A-51
2A-49
2A-48
2A-47
f1 (ppm)

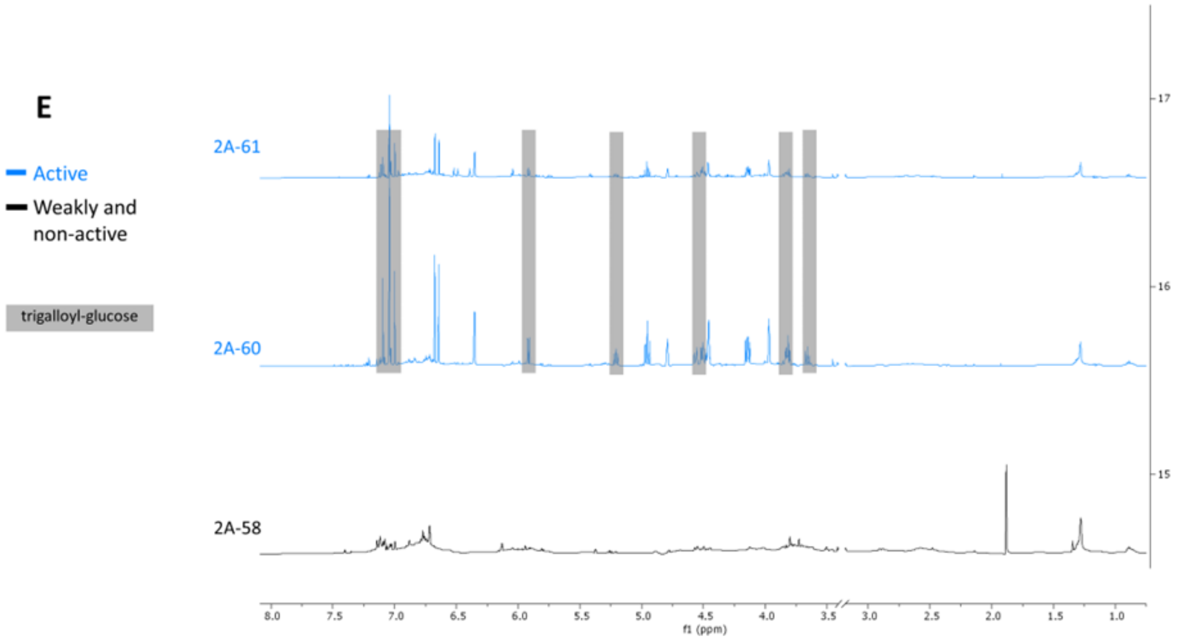

Supplementary Figure S6. ^{1}H NMR full spectra of the analyzed FR2-A subfractions (A) Summary of the active FRs (in blue) with respect to those adjacent weakly- or non-active (in black). The most relevant identified compounds are highlighted. (**B)** Spectra of 2A-29,2A-30,2A-31 with respect to adjacent 2A-27 and 2A-32. The active fractions exclusively contain fumaric acid (highlighted in red) and an unidentified pyrogallol-bearing compound (in yellow). **(C)** Spectra of 2A-35 and 2A-36 compared to adjacent 2A-33, 2A-39. The active fractions contain gallic acid (in blue) and myricetin hexoxide (in green), while the weakly active fraction 2A-39 contains galloyl-arbutin (in red). **(D)** Spectra of 2A-48 and 2A-49 compared to adjacent 2A-47 and 2A-51, the active fractions contain gallocatechin (in brown). In contrast, the non-active fraction 2A-47 contains catechin (in violet). (**E)** The spectra of 2A-60 and 2A-61 compared to 2A-58, the active fractions contain trigalloyl-glucose (in grey).

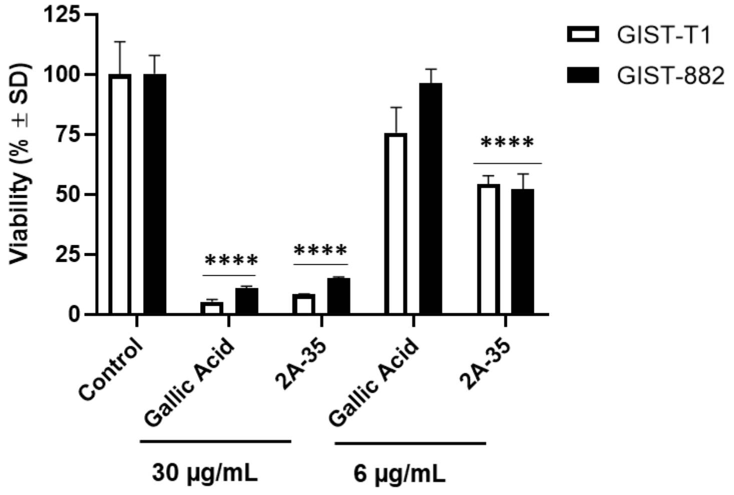

Supplementary Figure S7. Gallic acid in GIST-882 and GIST-T1 cells. Viability after gallic acid and 2A-35 treatments was measured by MTT assay. Both gallic acid and 2A-35 were used at 30 and 6 µg/mL. Viability is expressed in % ± SD with respect to control (DMSO). Viability was measured 24h after the MTT assay. Adjusted *P*-Value compared to DMSO: **** <0.0001 (one-way ANOVA and Dunnett's Multiple comparison test A representative experiment among three experimental replicates is shown.
